# Inhibition of HIV-1 Replication by Isoxazolidine and Isoxazole Sulfonamides

**DOI:** 10.1111/j.1747-0285.2010.00956.x

**Published:** 2010-05

**Authors:** Belinda Loh, Luciano Vozzolo, B James Mok, Chien Chi Lee, Richard J Fitzmaurice, Stephen Caddick, Ariberto Fassati

**Affiliations:** 1 Wohl Virion Centre, MRC Centre for Medical Molecular Virology, Division of Infection & ImmunityUCL, 46 Cleveland Street, London W1T 4JF, UK; 2 Department of Chemistry, University College London (UCL)20 Gordon Street, London WC1H 0AJ, UK

**Keywords:** gene expression, HIV-1, novel chemical entity, sulfonamides

## Abstract

Targeting host factors is a complementary strategy for the development of new antiviral drugs. We screened a library of isoxazolidine and isoxazole sulfonamides and found four compounds that inhibited HIV-1 infection in human CD4+ lymphocytic T cells with no toxicity at IC_90_ concentrations. Structure-activity relationship showed that benzyl sulfonamides and a halo-substituted aromatic ring on the heterocycle scaffold were critical for antiretroviral activity. The size and position of the incorporated halogen had a marked effect on the antiretroviral activity. The sulfonamide derivatives had no significant effect on HIV-1 entry, reverse transcription and integration but impaired a step necessary for activation of viral gene expression. This step was Tat-independent, strongly suggesting that the target is a cell factor. A virus partially resistant to the least potent compounds could be selected but could not be propagated in the long term, consistent with the possibility that HIV-1 may be less likely to develop resistance against drugs targeting some host factors. Here, we provide evidence that novel synthetic methods can be applied to develop small molecules with antiretroviral activity that target host factors important for HIV-1 replication.

An estimated 33.2 million people are infected with HIV-1, most of them living in Sub-Saharan Africa (a). There is no protective vaccine for HIV-1; thus, prevention and antiviral drugs are the only means to reduce virus transmission and replication. Several classes of antiretroviral drugs have been developed, which are effective at inhibiting different steps of the HIV-1 life cycle. The use of these drugs in combination, known as highly active antiretroviral therapy (HAART), has substantially decreased morbidity and mortality from HIV-1 infection (1).

A general problem is that HIV-1 rapidly acquires resistance, even to multiple drugs, by mutation, which leads to treatment failure in some patients (2–4). The drug-resistant viruses can still transmit and are not necessarily less fit than drug-naïve viruses (2,3). Furthermore, reservoirs of multi-drug-resistant HIV strains can persist undetected for many years (5). Hence, a secondary epidemic of drug-resistant virus may make currently available treatments less effective (4). For example, recent analysis on HIV-1 variants in the UK has shown an increase in the prevalence of drug-resistant HIV-1 strains (4,5). Furthermore, most existing antiretroviral drugs have considerable side–effects, and their availability in developing countries is limited. Thus, the development of new classes of antiretroviral drugs is important.

A complementary, yet little explored, pharmacological approach to inhibit virus replication is to target cellular factors necessary for infection (6). HIV-1, like all viruses, is a parasite that must exploit the cellular machinery to replicate. Thus, small molecules that prevent HIV-1 from interacting with such host factors may offer a valuable complement to the more classical approach based on inhibition of viral enzymes. The chemokine receptor antagonists are one example of antiviral drugs directed against cellular cofactors now in clinical use (7,8). A possible advantage of this approach lies in the lower mutation rate of cell factors compared to viral proteins; hence, viral mutants resistant to the inhibitors may be less likely to emerge.

The potential value of this approach for antiviral drug development is highlighted by three genome-wide, high throughput screens based on small interfering RNA (siRNA) technology, which identified several hundred host cell factors important for HIV replication (9–11). Although such studies are clearly important, it is difficult to predict which factors among the hundreds identified will be susceptible to modulation by small molecules and whether they represent a valid target for antiviral drug development.

Small molecules offer an alternative strategy for the identification of ‘druggable’ cellular targets necessary for HIV replication. Thus, by utilizing novel synthetic chemistry, it is possible to design interesting motifs, which are not generally available. If the chemistry is amenable to a library format, then medium high throughput screening can be used to identify small molecules with antiviral phenotypes that block specific steps of the viral life cycle. Once such molecules have been discovered, they can then be utilized for the identification of the targeted factor(s) (12,13), forming the basis for a more focused rational drug design aimed at finding lead compounds with greater antiviral potency and specificity. This experimental approach may also reveal new and unanticipated cellular pathways exploited by viruses to replicate.

Here, we describe the use of a new class of chemical entity, which has led to the identification of new molecules with antiretroviral activity that target host components important for specific steps of the viral life cycle. We describe the specific functional groups required for antiretroviral activity and provide evidence that HIV-1 may not easily escape the effect of small molecules targeting some host components.

## Methods and Materials

### Chemistry

Isoxazolidines **1a-h**, **1j**, **1m** and **1n** were prepared as previously described, and isoxazolidines **1i**, **1k**, **1l** and **1o–y** were prepared in an analogous fashion (14). Isoxazoles **2a–c**, **2j** and **2k** were prepared as previously described, and isoxazoles **2d–h** and **2l** were prepared in an analogous fashion (15).

(3R*,4R*)-3-(4-bromophenyl)-2-methyl-*N*-(4-methylbenzyl)-1,2-oxazolidine-4-sulfonamide (1i): R_f_ 0.31 (2:1 Et_2_O/petroleum ether 40–60 °C); mp 112–113 °C; IR (thin film) 3284, 2920, 2873, 1736, 1592, 1515, 1488, 1324, 1146, 686 cm^−1^; ^1^H NMR (500 MHz, CDCl_3_) δ 7.47 (2 H, d, *J*=8.2 Hz), 7.26 (2 H, d, *J*= 8.2 Hz), 7.07 (2 H, d, *J*= 7.7 Hz), 6.96 (2 H, d, *J*= 7.7 Hz), 5.04 (1 H, t, *J*= 5.5 Hz), 4.30 (1 H, dd, *J*= 9.7, 3.3 Hz), 4.15–4.19 (1 H, dd, *J*= 9.7, 8.5 Hz), 4.11 (1 H, dd, *J*= 13.8, 5.5 Hz), 3.97 (1 H, dd, *J*= 13.8, 5.5 Hz), 3.76–3.80 (2 H, m), 2.56 (3 H), 2.32 (3 H, s); ^13^C NMR (125 MHz, CDCl_3_) δ 138.04 (s), 136.15 (s), 133.22 (s), 132.16 (d), 129.93 (d), 129.37 (d), 128.21 (d), 122.80 (s), 73.55 (d), 72.10 (d), 66.97 (t), 47.14 (t), 42.76 (q), 21.22 (q); LRMS (EI) 426 (M^+•^, 7%), 424 (M^+•^, 6), 240 (100), 238 (95), 214 (14), 212 (16), 116 (43); HRMS (EI) calcd for C_18_H_21_BrN_2_O_3_S (M^+•^) 424.0450, observed 424.0447.

(3R*,4R*)-2-methyl-*N*-(4-methylbenzyl)-3-phenyl-1,2-oxazolidine-4-sulfonamide (1j): R_f_ 0.29 (2:1 Et_2_O/petroleum ether 40–60 °C); mp 96–98 °C; IR (thin film) 3300, 2922, 2856, 1589, 1456, 1414, 1319, 1144, 1034, 856 cm^−1^; ^1^H NMR (300 MHz, CDCl_3_) δ 7.48 (2 H, d, *J* = 8.0 Hz), 7.27 (2 H, d, *J* = 8.0 Hz), 7.08 (2 H, d, *J* = 7.7 Hz), 6.96 (2 H, d, *J* = 7.7 Hz), 4.91 (1 H, br t, *J* = 5.6 Hz), 4.29–4.33 (1 H, m), 4.09–4.21 (2 H, m), 3.98 (1 H, dd, *J* = 13.9, 5.6 Hz), 3.76–3.82 (2 H, m), 2.58 (3 H, s), 2.33 (3 H, s); ^13^C NMR (75 MHz, CDCl_3_) δ 138.0 (s), 136.1 (s), 133.1 (s), 132.1 (d), 129.8 (d), 129.5 (d), 128.0 (d), 122.8 (s), 73.6 (d), 73.5 (d), 66.9 (t), 47.1 (t), 42.7 (q), 21.2 (q); LRMS (EI) 426 (M^+^, 6%), 424 (M^+^, 8), 240 (63), 197 (28), 120 (100); HRMS (EI) calcd for C_18_H_21_BrN_2_O_3_S (M^+•^) 424.0456, observed 424.0450.

(3R*,4R*)-3-(2-bromophenyl)-2-methyl-*N*-(4-methylbenzyl)-1,2-oxazolidine-4-sulfonamide (1k): R_f_ 0.25 (2:1 Et_2_O/petroleum ether 40–60 °C); IR (thin film) 3251, 2978, 1737, 1433, 1328, 1148, 698 cm^−1^; ^1^H NMR (500 MHz, CDCl_3_) δ 7.59 (1 H, dd, *J* = 7.6, 1.1 Hz), 7.48 (1 H, d, *J* = 7.6 Hz), 7.38 (1 H, t, *J* = 7.6 Hz), 7.24 (1 H, t, *J* = 7.6 Hz), 7.01 (2 H, d, *J* = 7.7 Hz), 6.88 (2 H, d, *J* = 7.7 Hz), 4.60 (1 H, t, *J* = 5.7 Hz), 4.50 (1 H, d, *J* = 6.9 Hz), 4.46 (1 H, dd, *J* = 9.8, 3.6 Hz), 4.36 (1 H, dd, *J* = 9.8, 8.5 Hz), 4.13 (1 H, dd, *J* = 13.6, 5.7 Hz), 3.95 (1 H, td, *J* = 8.5, 3.6 Hz), 3.77 (1 H, dd, *J* = 13.6, 5.7 Hz), 2.64 (3 H, s), 2.30 (3 H, s); ^13^C NMR (125 MHz, CDCl_3_) δ 137.92 (s), 133.36 (d), 132.92 (s), 130.20 (d), 130.10 (d), 129.50 (d), 128.49 (s), 127.98 (d), 127.42 (d), 124.79 (s), 73.12 (d), 71.65 (d), 67.19 (t), 47.29 (t), 46.83 (q), 21.19 (q); LRMS (EI) 426 (M^+•^, 4%), 424 (M^+•^, 4), 240 (39), 197 (10), 120 (17), 105 (17); HRMS (EI) calcd for C_18_H_21_BrN_2_O_3_S (M^+•^) 424.0450, observed 424.0446.

(3R*,4R*)-3-(2-chlorophenyl)-2-methyl-*N*-(4-methylbenzyl)-1,2-oxazolidine-4-sulfonamide (1l): R_f_ 0.25 (2:1 Et_2_O/petroleum ether 40–60 °C); IR (thin film) 3283, 2922, 1737, 1438, 1328, 1148, 704 cm^−1^; ^1^H NMR (500 MHz, CDCl_3_) δ 7.49 (1 H, d, *J* = 7.3 Hz), 7.40 (1 H, dd, *J* = 7.4, 1.9 Hz), 7.33 (2 H, m), 7.05 (2 H, *J* = 7.9 Hz), 7.02 (2 H, d, *J* = 7.9 Hz), 4.54 (1 H, t, *J* = 5.8 Hz), 4.48 (1 H, d, *J* = 8.2 Hz), 4.45 (1 H, dd, *J* = 9.8, 3.8 Hz), 4.35 (1 H, dd, *J* = 9.8, 8.5 Hz), 4.13 (1 H, dd, *J* = 13.5, 5.7 Hz), 3.97 (1 H, td, *J* = 8.5, 3.8 Hz), 3.80 (1 H, dd, *J* = 13.5, 5.8 Hz), 2.63 (3 H, s), 2.30 (3 H, s); ^13^C NMR (125 MHz, CDCl_3_) δ 137.95 (s), 134.45 (s), 134.31 (s), 132.96 (s), 130.11 (d), 129.88 (d), 129.52 (d), 127.96 (d), 127.87 (d), 127.33 (d), 72.96 (d), 70.18 (d), 67.16 (t), 47.31 (t), 42.91 (q), 21.18 (q); LRMS (CI) 383 ((M+H)^+^, 34%), 381 ((M+H)^+^, 100), 197 (17), 195 (50), 120 (14); HRMS (CI) calcd for C_18_H_22_ClN_2_O_3_S ((M+H)^+^) 381.1039, observed 381.1045.

(3R*,4R*)-*N*-*tert*-butyl-3-(4-methoxyphenyl)-2-methyl-1,2-oxazolidine-4-sulfonamide (1o): R_f_ 0.18 (2:1 Et_2_O/petroleum ether 40–60 °C); mp 133–137 °C; IR (neat) 3280, 2961, 1614, 1516, 1435, 1305, 1252, 1138, 1010, 867 cm^−1^; ^1^H NMR (300 MHz, CDCl_3_) δ 7.37 (2 H, d, *J* = 8.7 Hz), 6.91 (2 H, d, *J* = 8.7 Hz), 4.57 (1 H, s), 4.41 (1 H, dd, *J* = 9.7, 3.8 Hz), 4.32 (1 H, dd, *J* = 9.7, 8.6 Hz), 3.97 (1 H, td, *J* = 8.0, 3.8 Hz), 3.81 (3 H, s), 3.72 (1 H, br d, *J* = 7.5 Hz), 2.60 (3 H, s), 1.08 (9 H, s); ^13^C NMR (75 MHz, CDCl_3_) δ 159.9 (s), 129.7 (d), 129.0 (s), 114.3 (d), 75.2 (d), 74.6 (d), 67.0 (t), 55.3 (q), 55.0 (s), 42.6 (q), 30.0 (q); LRMS (EI) 328 (M^+^, 21), 190 (100), 147 (32); HRMS (EI): calcd for C_15_H_24_N_2_O_4_S (M^+^) 328.1457, found 328.1449.

(3R*,4R*)-*N*-*tert*-butyl-3-(3-chlorophenyl)-2-methyl-1,2-oxazolidine-4-sulfonamide (1p): R_f_ 0.27 (2:1 Et_2_O/petroleum ether 40–60 °C); mp 117–119 °C; IR 3322, 2963, 2873, 1575, 1479, 1426, 1316, 1133, 1040, 986, 833, 797 cm^−1^; ^1^H NMR (300 MHz, CDCl_3_) δ 7.49 (1 H, s), 7.31–7.39 (3 H, m), 4.73 (1 H, s), 4.41 (1 H, dd, *J* = 9.7, 3.7 Hz), 4.31 (1 H, dd, *J* = 9.7, 8.4 Hz), 3.94 (1 H, td, *J* = 8.1, 3.7 Hz), 3.79 (1 H, d, *J* = 7.3 Hz), 2.64 (3 H, s), 1.12 (9 H, s); ^13^C NMR (75 MHz, CDCl_3_) δ 139.8 (s), 134.7 (s), 130.2 (d), 128.9 (d), 128.5 (d), 126.7 (d), 75.6 (d), 74.1 (d), 67.1 (t), 55.2 (s), 42.8 (q), 30.0 (q); LRMS (EI) 334 (M^+^, 4%), 332 (M^+^, 12), 194 (100), 115 (25); HRMS (EI): calcd for C_14_H_21_ClN_2_O_3_S (M^+^) 332.0961, found 332.0974.

(3R*,4R*)-*N*-*tert*-butyl-3-(4-chlorophenyl)-2-methyl-1,2-oxazolidine-4-sulfonamide (1q): R_f_ 0.24 (2:1 Et_2_O/petroleum ether 40–60 °C); mp 164–168 °C; IR (neat) 3267, 2921, 1493, 1307, 1141, 1091, 1012, 816 cm^−1^; ^1^H NMR (300 MHz, CDCl_3_) δ 7.42 (2 H, d, *J* = 8.5 Hz), 7.36 (2 H, d, *J* = 8.5 Hz), 4.85 (1 H, s), 4.40 (1 H, dd, *J* = 9.7, 3.7 Hz), 4.31 (1 H, dd, *J* = 9.7, 8.3 Hz), 3.93 (1 H, app. td, *J* = 7.9, 3.7 Hz), 3.78 (1 H, br d, *J* = 7.3 Hz), 2.61 (3 H, s), 1.12 (9 H, s); ^13^C NMR (75 MHz, CDCl_3_) δ 136.0 (s), 134.5 (s), 129.8 (d), 129.1 (d), 75.6 (d), 74.1 (d), 67.1 (t), 55.1 (s), 42.7 (q), 30.0 (q); LRMS (EI) 334 (M^+^, 10%), 332 (M^+^, 27), 194 (100), 174 (39), 151 (26), 115 (33); HRMS (EI): calcd for C_14_H_21_ClN_2_O_3_S (M^+•^) 332.0961, found 332.0976.

(3R*,4R*)-*N*-(2-hydroxyethyl)-2-methyl-3-phenyl-1,2-oxazolidine-4-sulfonamide (1r): R_f_ 0.15 (2:1 EtOAc/petroleum ether 40–60 °C); IR 3480, 3279, 2921, 1456, 1311, 1144, 1039, 934, 887 cm^−1^; ^1^H NMR (300 MHz, CDCl_3_) δ 7.33–7.47 (5 H, m), 5.53 (1 H, t, *J* = 6.2 Hz), 4.38 (1 H, dd, *J* = 9.9, 4.3 Hz), 4.43 (1 H, dd, *J* = 9.9, 7.8 Hz), 4.08 (1 H, td, *J* = 7.4, 4.3 Hz), 3.82 (1 H, br d, *J* = 6.4 Hz), 3.43–3.56 (2 H, m), 3.08 (1 H, ddt, *J* = 12.6, 6.2, 3.9 Hz), 2.84–2.96 (1 H, m), 2.69 (1 H, br s), 2.62 (3 H, s); ^13^C NMR (75 MHz, CDCl_3_) δ 136.8 (s), 129.0 (d), 128.8 (d), 128.3 (d), 74.4 (d), 73.1 (d), 67.0 (t), 61.6 (t), 45.4 (t), 42.7 (q); LRMS (EI) 286 (M^+•^, 11%), 160 (100), 117 (48); HRMS (EI): calcd for C_12_H_18_N_2_O_4_S (M^+•^) 286.0987, found 286.0981.

(3R*,4R*)-*N*-(2-hydroxyethyl)-2-methyl-3-(naphthalen-2-yl)-1,2-oxazolidine-4-sulfonamide (1s): R_f_ 0.33 (9:1 CHCl_3_/MeOH); IR 3457, 3284, 2923, 1509, 1435, 1314, 1144, 1040, 955 cm^−1^; ^1^H NMR (300 MHz, CDCl_3_) δ 7.93 (1 H, s), 7.81–7.87 (3 H, m), 7.57 (1 H, dd, *J* = 8.6, 1.5 Hz), 7.49 (2 H, dt, *J* = 9.5, 3.6 Hz), 5.69 (1 H, br t, *J* = 5.8 Hz), 4.43 (1 H, dd, *J* = 10.0, 4.0 Hz), 4.38 (1 H, dd, *J* = 10.0, 7.6 Hz), 4.17 (1 H, app. td, *J* = 7.6, 4.0 Hz), 4.02 (1 H, br d, *J* = 6.6 Hz), 3.36–3.49 (2 H, m), 2.82–3.08 (3 H, m), 2.65 (3 H, s); ^13^C NMR (75 MHz, CDCl_3_) δ 134.2 (s), 133.4 (s), 133.2 (s), 128.9 (d), 128.0 (2 × d), 127.8 (d), 126.6 (2 × d), 125.1 (d), 74.5 (d), 73.1 (d), 67.1 (t), 61.5 (t), 45.4 (t), 42.8 (q); LRMS (EI) 336 (M^+•^, 24%), 210 (100), 184 (23), 167 (58), 152 (33), 127 (20); HRMS (EI): calcd for C_16_H_20_N_2_O_4_S (M^+^) 336.1144, found 336.1138.

(3R*,4R*)-3-(5-bromofuran-2-yl)-2-methyl-*N*-(4-methylbenzyl)-1,2-oxazolidine-4-sulfonamide (1t): R_f_ 0.27 (2:1 Et_2_O/petroleum ether 40–60 °C); IR (thin film) 3142, 2919, 1441, 1320, 1141, 1020, 901, 793 cm^−1^; ^1^H NMR (300 MHz, CDCl_3_) δ 7.14 (2 H, d, *J* = 8.0 Hz), 7.08 (2 H, d, *J* = 8.0 Hz), 6.37 (1 H, d, *J* = 3.5 Hz), 6.31 (1 H, d, *J* = 3.5 Hz), 4.81 (1 H, br s), 4.33 (1 H, dd, *J* = 9.1, 3.2 Hz), 4.05–4.24 (4 H, m), 3.89 (1 H, br s), 2.67 (3 H, s), 2.34 (3 H, s); ^13^C NMR (75 MHz, CDCl_3_) δ 150.6 (s), 138.1 (s), 133.1 (s), 129.6 (d), 128.0 (d), 123.0 (s), 113.8 (d), 112.6 (d), 69.4 (d), 67.5 (d), 66.8 (t), 47.2 (t), 42.8 (q), 21.2 (q); LRMS (EI) 416 (M^+•^, 7%), 414 (M^+^, 7), 230 (100), 187 (52), 159 (22), 120 (95); HRMS (EI) calcd for C_16_H_19_BrN_2_O_4_S (M^+^) 414.0194, observed 414.0206.

(3R*,4R*)-3-(4-fluorophenyl)-2-methyl-*N*-(4-methylbenzyl)-1,2-oxazolidine-4-sulfonamide (1u): R_f_ 0.29 (2:1 Et_2_O/petroleum ether 40–60 °C); mp 129–132 °C; IR (thin film) 3284, 2923, 2874, 1606, 1510, 1324, 1146, 841 cm^−1^; ^1^H NMR (500 MHz, CDCl_3_) δ 7.37 (2 H, d, *J* = 8.5 Hz), 7.07 (2 H, d, *J* = 8.0 Hz), 7.03 (2 H, d, *J* = 8.5 Hz), 6.97 (2 H, d, *J* = 8.0 Hz), 4.93 (1 H, t, *J* = 5.3 Hz), 4.31 (1 H, dd, *J* = 9.7, 3.3 Hz), 4.19 (1 H, dd, *J* = 9.7, 7.8 Hz), 4.11 (1 H, dd, *J* = 13.5, 5.3 Hz), 3.96 (1H, dd, *J* = 13.5, 5.3 Hz), 3.79–3.84 (2 H, m), 2.57 (3 H, s), 2.31 (3 H, s); ^13^C NMR (125 MHz, CDCl_3_) δ 162.94 (s, *J*_CF_ = 248.6 Hz), 138.02 (s), 133.26 (s), 132.79 (s, *J*_CF_ = 2.9 Hz), 129.95 (d, *J*_CF_ = 8.6 Hz), 129.56 (d), 128.02 (d), 115.98 (d, *J*_CF_ = 22.1 Hz), 73.58 (d), 72.16 (d), 66.93 (t), 74.63 (t), 42.69 (q), 21.17 (q); LRMS (EI) 364 (M^+^, 8%), 178 (100), 135 (30); HRMS (EI) calcd for C_18_H_21_FN_2_O_3_S (M^+^) 364.1251, observed 364.1246.

(3R*,4R*)-3-(4-chlorophenyl)-2-methyl-*N*-(4-methylbenzyl)-1,2-oxazolidine-4-sulfonamide (1v): R_f_ 0.29 (2:1 Et_2_O/petroleum ether 40–60 °C); IR (thin film) 3285, 2922, 2874, 1736, 1599, 1515, 1492, 1323, 1145, 736 cm^−1^; ^1^H NMR (500 MHz, CDCl_3_) δ 7.31–7.34 (4 H, m), 7.07 (2 H, d, *J*= 8.0 Hz), 6.96 (2 H, d, *J*= 8.0 Hz), 5.00 (1 H, t, *J*= 5.5 Hz), 4.30 (1 H, dd, *J*= 9.7, 3.3 Hz), 4.18 (1 H, dd, *J*= 9.7, 7.9 Hz), 4.11 (1 H, dd, *J*= 13.8, 5.6 Hz), 3.97 (1H, dd, *J*= 13.8, 5.5 Hz), 3.77–3.82 (2 H, m), 2.57 (3 H, s), 2.32 (3 H, s); ^13^C NMR (125 MHz, CDCl_3_) δ 137.93 (s), 135.62 (s), 134.63 (s), 133.23 (s), 129.56 (d), 129.21 (d), 128.89 (d), 128.02 (d), 73.59 (d), 72.16 (d), 66.97 (t), 47.51 (t), 42.76 (q), 21.20 (q); LRMS (EI) 382 (M^+•^, 2%), 380 (M^+^, 5), 196 (38), 194 (100), 178 (25), 151 (42), 120 (51); HRMS (EI) calcd for C_18_H_21_ClN_2_O_3_S (M^+^) 380.0956, observed 380.0963.

(3R*,4R*)-3-(4-iodophenyl)-2-methyl-*N*-(4-methylbenzyl)-1,2-oxazolidine-4-sulfonamide (1w): R_f_ 0.27 (2:1 Et_2_O/petroleum ether 40–60 °C); mp 132–134 °C; IR (thin film) 3283, 2956, 2899, 1737, 1515, 1327, 1148 cm^−1^; ^1^H NMR (500 MHz, CDCl_3_) δ 7.68 (2 H, d, *J* = 8.3 Hz), 7.13 (2 H, d, *J* = 8.3 Hz), 7.07 (2 H, d, *J* = 7.9 Hz), 6.95 (2 H, d, *J* = 7.9 Hz), 4.97 (1 H, t, *J* = 5.6 Hz), 4.30 (1 H, d, *J* = 9.7 Hz), 4.17 (1 H, dd, *J* = 9.7, 3.3 Hz), 4.10 (1 H, dd, *J* = 13.8, 5.6 Hz), 3.96 (1 H, dd, *J* = 13.8, 5.6 Hz), 3.74–3.81 (2 H, m,), 2.56 (3 H, s), 2.32 (3 H, s); ^13^C NMR (125 MHz, CDCl_3_) δ 138.13 (d), 136.83 (s), 132.89 (s), 129.90 (d), 129.60 (d), 128.90 (s), 128.21 (d), 94.55 (s), 73.74 (d), 73.51 (d), 66.97 (t), 47.51 (t), 42.80 (q), 21.26 (q); LRMS (EI) 472 (M^+•^, 5%), 286 (100), 260 (12), 120 (30); HRMS (EI) calcd for C_18_H_21_IN_2_O_3_S (M^+^) 472.0312, observed 472.0309.

(3R*,4R*)-3-(2-fluorophenyl)-2-methyl-*N*-(4-methylbenzyl)-1,2-oxazolidine-4-sulfonamide (1x): R_f_ 0.29 (2:1 Et_2_O/petroleum ether 40–60 °C); IR (thin film) 3286, 2924, 2877, 1737, 1617, 1588, 1518, 1327, 1146, 844 cm^−1^; ^1^H NMR (500 MHz, CDCl_3_) δ 7.41 (1 H, t, *J* = 7.1 Hz), 7.33–7.38 (1 H, m), 7.18 (1 H, d, *J* = 7.1 Hz), 7.07 (1H, t, *J* = 7.1 Hz), 7.04 (2 H, d, *J* = 7.9 Hz), 6.95 (2 H, d, *J* = 7.9 Hz), 4.85 (1 H, t, *J* = 5.3 Hz), 4.38 (1 H, dd, *J* = 9.7, 3.5 Hz), 4.27 (1 H, dd, *J* = 9.7, 8.3 Hz), 4.14 (1 H, dd, *J* = 13.7, 5.3 Hz), 4.11 (1 H, d, *J* = 3.5 Hz), 4.02 (1 H, d, *J* = 3.5 Hz), 3.94 (1 H, dd, *J* = 13.7, 5.2 Hz), 2.60 (3 H, s), 2.30 (3 H, s); ^13^C NMR (125 MHz, CDCl_3_) δ 161.08 (s, *J*_CF_ = 248.6 Hz), 137.89 (s), 113.94 (s), 130.50 (d, *J*_CF_ = 8.6 Hz), 129.93 (d, *J*_CF_ = 3.8 Hz), 129.51 (d), 127.99 (d), 124.99 (d, *J*_CF_ = 3.8 Hz), 123.70 (s, *J*_CF_ = 10.6 Hz), 116.16 (d, *J*_CF_ = 22.1 Hz), 72.23 (d), 68.12 (d), 67.08 (t), 47.51 (t), 42.90 (q), 21.16 (q); LRMS (CI) 365 ((M+H)^+^, 100%), 178 (24), 120 (4); HRMS (CI) calcd for C_18_H_22_FN_2_O_3_S ((M+H)^+^) 365.1335, observed 365.1345.

(3R*,4R*)-3-(2-iodophenyl)-2-methyl-*N*-(4-methylbenzyl)-1,2-oxazolidine-4-sulfonamide (1y): R_f_ 0.27 (2:1 Et_2_O/petroleum ether 40–60 °C); IR (thin film) 3310, 2922, 2874, 1736, 1515, 1432, 1326, 1147 cm^−1^; ^1^H NMR (500 MHz, CDCl_3_) δ 7.87 (1 H, d, *J* = 7.9 Hz), 7.40 (2 H, d, *J* = 7.9 Hz), 7.06–7.10 (1 H, m), 7.01 (2 H, *J* = 7.9 Hz), 6.89 (2 H, d, *J* = 7.9 Hz), 4.70 (1 H, t, *J* = 5.8 Hz), 4.44 (1 H, d, *J* = 9.8 Hz), 4.38 (1 H, d, *J* = 7.1 Hz), 4.34 (1 H, dd, *J* = 9.8, 8.3 Hz), 4.13 (1 H, dd, *J* = 13.5, 5.9 Hz), 3.91 (1 H, t, *J* = 8.2 Hz), 3.78 (1 H, dd, *J* = 13.5, 5.8 Hz), 2.64 (3 H, s), 2.30 (3 H, s); ^13^C NMR (125 MHz, CDCl_3_) δ 140.07 (d), 139.14 (s), 137.89 (s), 132.96 (s), 130.49 (d), 129.79 (d), 129.50 (d), 129.26 (d), 128.03 (d), 101.18 (s), 76.85 (d), 73.43 (d), 67.24 (t), 47.28 (t), 42.55 (q), 21.20 (q); LRMS (CI) 473 ((M+H)^+^, 100%), 287 (52), 120 (20), 105 (18); HRMS (CI) calcd for C_18_H_22_IN_2_O_3_S ((M+H)^+^) 473.0395, observed 473.0403.

**3-(3-chlorophenyl)-*N*-(4-methylbenzyl)-1,2-oxazole-5-sulfonamide (2d):** R_f_ 0.08 (20% Et_2_O/petroleum ether 40–60 °C); mp 101–104 °C; IR (thin film) 3363, 3055, 2985, 1575, 1172 cm^−1^; ^1^H NMR (300 MHz, CDCl_3_) δ 7.74 (1 H, s), 7.62 (1 H, d, *J* = 6.7 Hz), 7.46 (1 H, t, *J* = 6.7 Hz), 7.41 (1 H, d, *J* = 6.9 Hz), 7.14 (2 H, d, *J* = 7.0 Hz), 7.08 (2 H, d, *J* = 7.0 Hz), 6.91 (1 H, s), 5.43 (1 H, br s), 4.34 (2 H, s), 2.22 (3 H, s); ^13^C NMR (75 MHz, CDCl_3_) δ 166.9 (s), 161.4 (s), 138.5 (s), 135.2 (s), 132.1 (s), 130.9 (d), 130.5 (d), 129.5 (d), 129.0 (s), 128.1 (d), 127.0 (d), 125.0 (d), 105.8 (d), 47.4 (t), 21.0 (q); LRMS (FAB) 365 ((M+H)^+^, 13%), 363 ((M+H)^+^, 40), 289 (12), 154 (100); HRMS (FAB) calcd for C_17_H_16_ClN_2_O_3_S ((M+H)^+^) 363.0570, observed 363.0576.

**3-(2-chlorophenyl)-*N*-(4-methylbenzyl)-1,2-oxazole-5-sulfonamide (2e):** R_f_ 0.06 (20% Et_2_O/petroleum ether 40–60 °C); mp 84–86 °C; IR (thin film) 3300, 3055, 1353, 1170 cm^−1^; ^1^H NMR (300 MHz, CDCl_3_) δ 7.68 (1 H, dd, *J* = 7.3, 1.9 Hz), 7.52 (1 H, dd, *J* = 7.3, 1.9 Hz), 7.44 (1 H, td, *J* = 7.3, 2.1 Hz), 7.37 (1 H, td, *J* = 7.4, 2.0 Hz), 7.15 (2 H, d, *J* = 8.1 Hz), 7.15 (1 H, s), 7.08 (2 H, d, *J* = 8.1 Hz), 5.65 (1 H, t, *J* = 5.9 Hz), 4.35 (2 H, d, *J* = 6.0 Hz), 2.25 (3 H, s); ^13^C NMR (75 MHz, CDCl_3_) δ 165.9 (s), 161.1 (s), 138.0 (s), 132.9 (s), 132.3 (s), 131.7 (d), 131.0 (d), 130.6 (d), 129.5 (d), 128.0 (d), 127.3 (d), 126.6 (s), 108.8 (d), 47.4 (t), 21.0 (q); LRMS (FAB) 365 ((M+H)^+^, 30%), 363 ((M+H)^+^, 86), 307 (34), 289 (18), 165 (100); HRMS (FAB) calcd for C_17_H_16_ClN_2_O_3_S ((M+H)^+^) 363.0570, observed 363.0573.

**3-(4-bromophenyl)-*N*-(4-methylbenzyl)-1,2-oxazole-5-sulfonamide (2f):** R_f_ 0.06 (20% Et_2_O/petroleum ether 40–60 °C); mp 156–158 °C; IR (thin film) 3373, 3053, 2985, 1170 cm^−1^; ^1^H NMR (300 MHz, CDCl_3_) δ 7.63 (4 H, s), 7.15 (2 H, d, *J* = 8.1 Hz), 7.09 (2 H, d, *J* = 8.1 Hz), 6.93 (1 H, s), 5.25 (1 H, t, *J* = 5.9 Hz), 4.34 (2 H, d, *J* = 5.9 Hz), 2.24 (3 H, s); ^13^C NMR (75 MHz, CDCl_3_) δ 166.1 (s), 161.3 (s), 138.2 (s), 132.5 (d), 132.2 (s), 129.5 (d), 128.3 (d), 128.0 (d), 125.0 (s), 114.7 (s), 105.7 (d), 47.5 (t), 21.0 (q); LRMS (FAB) 409 ((M+H)^+^, 26%), 407 ((M+H)^+^, 26), 307 (17), 286 (33), 154 (100); HRMS (FAB) calcd for C_17_H_16_BrN_2_O_3_S (M+H)^+^ 407.0065, observed 407.0048.

**3-(3-bromophenyl)-*N*-(4-methylbenzyl)-1,2-oxazole-5-sulfonamide (2g):** R_f_ 0.06 (20% Et_2_O/petroleum ether 40–60 °C); mp 101–103 °C; IR (thin film) 3367, 3055, 2985, 1326, 1172 cm^−1^; ^1^H NMR (300 MHz, CDCl_3_) δ 7.89 (1 H, s), 7.61–7.68 (2 H, m), 7.36 (1 H, t, *J* = 7.8 Hz), 7.14 (2 H, d, *J* = 7.8 Hz), 7.08 (2 H, d, *J* = 7.8 Hz), 6.90 (1 H, s), 5.47 (1 H, br s), 4.34 (2 H, s), 2.22 (3 H, s); ^13^C NMR (75 MHz, CDCl_3_) δ 166.9 (s), 161.3 (s), 138.2 (s), 133.8 (d), 132.1 (s), 130.7 (d), 129.9 (d), 129.5 (d), 129.3 (s), 128.1 (d), 125.5 (d), 123.2 (s), 105.7 (d), 47.4 (t), 21.0 (q); LRMS (FAB) 409 ((M+H)^+^, 44%), 407 ((M+H)^+^, 46), 307 (15), 154 (100); HRMS (FAB) calcd for C_17_H_16_BrN_2_O_3_S ((M+H)^+^) 407.0065, observed 407.0060.

**3-(2-bromophenyl)-*N*-(4-methylbenzyl)-1,2-oxazole-5-sulfonamide (2h):** R_f_ 0.10 (20% Et_2_O/petroleum ether 40–60 °C); mp 102–104 °C; IR (thin film) 3365, 2052, 2985, 1170 cm^−1^; ^1^H NMR (300 MHz, CDCl_3_) δ 7.70 (1 H, dd, *J* = 7.6, 1.8 Hz), 7.61 (1 H, dd, *J* = 7.6, 1.8 Hz), 7.44 (1H, td, *J* = 7.5, 1.8 Hz), 7.36 (1H, td, *J* = 7.6, 2.1 Hz), 7.15 (1 H, s), 7.15 (2 H, d, *J* = 8.2 Hz), 7.10 (2 H, d, *J* = 8.2 Hz), 5.47 (1 H, t, *J* = 5.8 Hz), 4.36 (2 H, d, *J* = 5.8 Hz), 2.29 (3 H, s); ^13^C NMR (75 MHz, CDCl_3_) δ 165.7 (s), 162.5 (s), 138.1 (s), 133.8 (d), 132.3 (s), 131.8 (d), 131.4 (d), 129.5 (d), 128.7 (s), 122.2 (s), 127.9 (d), 127.8 (d), 108.9 (d), 47.4 (t), 21.1 (q); LRMS (FAB) 409 ((M+H)^+^, 91%), 407 ((M+H)^+^, 100), 219 (30), 154 (36); HRMS (FAB) calcd for C_17_H_16_BrN_2_O_3_S ((M+H)^+^) 407.0065, observed 407.0061.

**3-(2-fluorophenyl)-*N*-(4-methylbenzyl)-1,2-oxazole-5-sulfonamide (2j):** R_f_ 0.08 (20% Et_2_O/petroleum ether 40–60 °C); mp 111–113 °C; IR (thin film) 3365, 3055, 2985, 1170 cm^−1^; ^1^H NMR (300 MHz, CDCl_3_) δ 7.96 (1 H, td, *J* = 5.8, 1.7 Hz), 7.50–7.61 (1 H, m), 7.18–7.30 (2 H, m), 7.15 (2 H, d, *J* = 8.9 Hz), 7.12 (1 H, s), 7.09 (2 H, d, *J* = 8.9 Hz), 5.30 (1 H, t, *J* = 5.9 Hz), 4.34 (2 H, d, *J* = 5.9 Hz), 2.24 (3 H, s); ^13^C NMR (125 MHz, CDCl_3_) δ 166.44 (s), 160.1 (s, *J*_CF_ = 252.6 Hz), 157.9 (s), 138.2 (s), 132.70 (s, *J*_CF_ = 8.5 Hz), 132.2 (s), 129.5 (d), 129.0 (d), 128.0 (d), 125.3 (d), 124.8 (d, *J*_CF_ = 3.8 Hz), 116.6 (d, *J*_CF_ = 21.4 Hz), 108.2 (d, *J*_CF_ = 9.7 Hz), 47.4 (t), 21.0 (q); LRMS (FAB) 347 ((M+H)^+^, 100%), 251 (20), 154 (58); HRMS (FAB) calcd for C_17_H_16_FN_2_O_3_S ((M+H)^+^) 347.0865, observed 347.0869.

**3-(2-iodophenyl)-*N*-(4-methylbenzyl)-1,2-oxazole-5-sulfonamide (2k):** R_f_ 0.2 (20% Et_2_O/petroleum ether 40–60 °C); mp 115–117 °C; IR (thin film) 3297, 1354, 1165 cm^−1^; ^1^H NMR (300 MHz, CDCl_3_) δ 8.02 (1 H, dd, *J* = 7.5, 1.1 Hz), 7.52 (1 H, td, *J* = 7.6, 1.1 Hz), 7.43 (1 H, dd, *J* = 7.5, 1.3 Hz), 7.24 (1 H, td, *J* = 7.8, 1.1 Hz), 7.14 (2 H, d, *J* = 8.3 Hz), 7.09 (2 H, d, *J* = 8.3 Hz), 7.01 (1 H, s), 6.82 (1 H, t, *J* = 5.9 Hz), 4.28 (2 H, d, *J* = 5.9 Hz), 2.24 (3 H, s); ^13^C NMR (75 MHz, CDCl_3_) δ 167.2 (s), 165.9 (s), 141.3 (d), 138.6 (s), 134.4 (s), 134.0 (s), 132.9 (d), 131.9 (d), 130.2 (d), 129.7 (d), 129.0 (d), 109.7 (d), 97.0 (s), 47.7 (t), 21.3 (q); LRMS (EI) 454 (M^+•^, 22), 336 (20), 270 (44), 242 (55), 229 (86), 118 (86), 104 (100); HRMS (EI) calcd for C_17_H_16_N_2_O_3_S (M^+•^) 453.9846, observed 453.9864.

### Cells and viruses

HeLa, HT1080 and 293T cells were grown in Dulbecco’s modified Eagle’s medium (DMEM) supplemented with 10% fetal calf serum (FCS) and 2 mm glutamine at 37 °C in 5% CO_2_. Jurkat, SupT1 and C8166 cells were grown in RPMI medium supplemented with 10% FCS at 37 °C in 10% CO_2_. The HIV-1 vectors were made and purified as described previously (16–18). HIV-1 NL4.3 (19) was produced by Fugene transfection into 293T cells, and supernatant containing viral particles was collected 48 h post transfection. Reverse transcriptase (RT) activity was measured by the Lenti-RT™ Activity Assay (Cavidi Tech, Uppsala, Sweden) following the manufacturer’s instructions.

### Infection assays

Approximately 4 × 10^5^ adherent cells or 0.5 × 10^6^ lymphocytic cells were plated in 24-well plates in 500 μL of media incubated for 6 h with the compounds and infected at a MOI of ∼0.05 using a VSV-G-pseudotyped HIV-1 vector. Infected cells were incubated with the compounds for 24 h, washed and analyzed by FACS. Total DNA was extracted from an aliquot of infected cells and analyzed by Taqman qPCR. In some experiments, one aliquot of infected cells was analyzed 24 h post infection and another aliquot 1 week later. For infection with wild-type HIV-1 NL4.3, 0.5 × 10^6^ lymphocytic cells were plated into 24-well plates in 500 μL of medium and cultured in the presence of the compounds for 6 h. The culture was transferred to 96 U-well plates in 100 μL aliquots and infected at MOI of 0.1. Cells were grown for 48–72 h, washed once in serum-free medium, fixed in 50% methanol–50% acetone for 2 min at −20 °C and immunostained as previously described (20) using anti-HIV-1 p24 Ab (38:96K and EF7 at 1:1 ratio, AIDS Reagent Program) and secondary anti-mouse Abs conjugated to β-galactosidase (Southern Biotechnology, Birmingham, AL, USA) diluted 1/400. Alternatively, HIV-infected cells were fixed for 20 min at room temperature in 100 μL 4% paraformaldehyde in PBS, washed and permeabilized in 100 μL of cytofix/cytoperm solution (BD Biosciences, San Jose, CA, USA) for 30 min at 4 °C. The same primary anti-p24 Ab was detected by an anti-mouse immunoglobulin FITC conjugated, diluted 1/200, and cells were analyzed by FACS. Macrophages immunostained for p24 were counted using an MRX TC Revelation microplate reader (Dynex Technologies, Chantilly, VA, USA).

### Cell toxicity assays

For cell cycle profiling, ∼2 × 10^6^ cells were resuspended in 50 μL PBS + 3% FCS and fixed by addition of 1 mL cold 80% ethanol and incubation at 4 °C for 30 min. Cells were resuspended in 0.5 mL PBS + 0.25% (v/v) NP-40 (IGEPAL CA-630) (Sigma, Gillingham, Dorset, UK), 2 U of DNAse-free RNAse A (Roche, Welwyn Garden City, UK), incubated for 30 min at 37 °C and centrifuged. The pellet was resuspended in 400 μL PBS + 0.1 μg propidium iodide (Sigma) and analyzed by FACS. Cell viability was analyzed with the LIVE/DEAD kit (Molecular Probes, Paisley, UK) following the manufacturers’ instructions or by trypan blue staining.

### Taqman qPCR and RT-qPCR

Approximately 1 × 10^6^ cells were washed twice in PBS, and total DNA was extracted with the Qiamp® DNA Minikit (Qiagen, Crawley, UK). Quantitative PCRs were carried out as previously described (18,21) in 25 μL volume containing 100 ng total DNA using an ABI Prism® 7000 Sequence Detection System (SDS). For mRNA quantification, nucleic acids were extracted from 1 × 10^7^ SupT1 cells using the RNeasy Mini Kit (Qiagen) according to the manufacturer’s instructions. Samples were treated with 2 U/μg nucleic acids ReQ1 DNAse in 1× buffer (Promega, Southampton, UK) for 30 min at 37 °C, then 2 mm EGTA were added to stop the reaction, and samples were incubated at 60 °C for 20 min. The Superscript III kit (Invitrogen, Paisley, UK) was used for first-strand cDNA synthesis by random hexamers following the manufacturer’s instructions. Control samples were incubated in parallel without RT. TaqMan quantitative PCR was performed in an ABI Prism 7000 thermocycler with the GFP primers/probe set (18), 2LTR (Long Terminal Repeat) circular DNA primer set (21) and the GADPH RNA primers/probe set GADPH-F GGCTGAGAACGGGAAGCTT, GADPH-RC AGGGATCTCGCTCCTGGAA, probe FAM-TCATCAATGGAAATCCCATCACCA-TAMRA.

### Sequencing of parental and resistant viruses

The sequences of parental and resistant NL4-3 viruses were obtained by PCR amplification of 15 different overlapping segments covering the entire viral genome. Total DNA was extracted from chronically infected cells using the Qiagen DNA extraction Kit. The PCR was performed in 50 μL containing 1× buffer (Promega), 33 pmol each primer, 0.5 μL Pfu DNA polymerase and 100 μm dNTPs using four different annealing temperatures ranging from 45 to 65 °C to select the optimal conditions for each set of primers. The PCR products were resolved on a 1% agarose gel and visualized by ethidium bromide. The different sets of primers are specified below.

**Table:** 

Primer	Sequence (5′-3′)	T_AN_ (°C)
NL4-3 1F NL4-3 1R	TGG AAG GGC TAA TTT GGT CCC GAG AGA TCT CCT CTG GCT TTA CT	65
NL4-3 2F NL4-3 2R	GCC CGT CTG TTG TGT GAC TCT G GCA CTC TAT CCC ATT CTG CAG C	60
NL4-3 3F NL4-3 3R	CAT TAT CAG AAG GAG CCA CCC CTT CTT TGC CAC AAT TGA AAC A	65
NL4-3 4F NL4-3 4R	ATG ATG ACA GCA TGT CAG GGA G CAA CAG ATG TTG TCT CAG TTC CTC	60
NL4-3 5F NL4-3 5R	CAG ATG ATA CAG TAT TAG AAG CAA CAG ATG TTG TCT CAG TTC CTC	45
NL4-3 6F NL4-3 6R	GGA TCA CCA GCA ATA TTC CAG TGT CAG TTA CAT ATC CTG CTT TTC C	55
NL4-3 7F NL4-3 7R	CTG GAT TCC TGA GTG GGA GTT T GAG GAA GTA TGC TGT TTC TTG CCC	55
NL4-3 8F NL4-3 8R	GTA GAC TGT AGC CCA GGA ATA TGG TAC TAA TTT AGC ATC CCC TAG TGG	55
NL4-3 9F NL4-3 9R	GTA GAC AGG ATG AGG ATT AAC ACA TGG GAC TGT TCT GAT GAG CTC TTC GTC G	60
NL4-3 10F NL4-3 10R	GTC AGC CTA AAA CTG CTT GTA CCA GCA CCT TAT CTC TTA TGC TTG TGC TG	50
NL4-3 11F NL4-3 11R	GAC TAG GTA GAA CAG ATG CAT GAG GA GTC CCC TCC TGA GGA TTG CTT AAA G	60
NL4-3 12F NL4-3 12R	GAC CAG GGA GAG CAT TTG TTA CAA TAG CCT CAA TAG CCC TCA GCA AAT TGT TC	60
NL4-3 13F NL4-3 13R	GAG CAG TGG GAA TAG GAG CTT TG CTA ATC GAA TGG ATC TGT CTC TGT CTC	60
NL4-3 14F NL4-3 14R	CTT TCT ATA GTG AAT AGA GTT AGG CAG GG CAT TGG TCT TAA AGG TAC CTG AGG TG	55
NL4-3 15F NL4-3 15R	GGA GCA ATC ACA AGT AGC AAT ACA GC TGG AAT GCA GTG GCG CGA TCT	65

3′-A overhangs were added by Taq-polymerase on PCR samples by one additional PCR cycle to enable direct cloning of the PCR product into the Topo vector, following the manufacturer’s instructions (TOPO TA cloning kit; Invitrogen). The plasmids carrying the fragments of interest were purified from overnight bacterial cultures using the Qiagen Miniprep kit. Sequencing was carried out by the UCL Sequencing Service using the M13 primers.

## Results

### Antiretroviral activity of isoxazolidine sulfonates and sulfonamides

We have synthesized novel series of isoxazolidines bearing two points of diversity; at the sulfonate/sulfonamide, R^1^, and at the C-3 position of the heterocyclic scaffold, R^2^. Initially, we tested a small collection of 19 compounds with a range of substituents at R^1^ and R^2^ for their ability to inhibit HIV infection and replication (Table 1). The initial screen was performed using a HIV-1 based vector that was pseudotyped with the vesicular stomatitis virus G-protein (VSV-G) and expressed the green fluorescent protein (GFP) (16). Human CD4+ T-lymphoid SupT1 cells were preincubated with the compounds for 6 h, infected with the HIV-1 vector at a multiplicity of infection (MOI) of ∼0.05 and analyzed by flow cytometry 24 h later. The percentage of GFP+ cells was then plotted against the compound dose. This assay allowed rapid screen of the compound library and an accurate titration of the dose-response curve. Compounds with IC_90_ values lower than 150 μm were considered to be inhibitors of HIV infection. Of the library-screened, three compounds, **1i**, **1k** and **1l,** had significant antiretroviral activity indicating the importance of aromatic groups in both the sulfonamide motif and directly appended to the heterocyclic scaffold. Compounds bearing sulfonate esters in the C-4 position of the isoxazolidine scaffold were in general only moderately active. Isoxazolidine **1f**, bearing an allyl sulfonamide, was observed to be ineffective and was hence included in all other experiments as a negative control (Table 1). The most active compounds, **1i**, **1k** and **1l**, were then retested in the human CD4+ T-lymphocytic C8166 cells, which were infected with wild-type NL4.3 HIV-1. Cells were pretreated with the compounds for 6 h and infected in the presence of the compounds for 24 h. Media was changed, and cells analyzed by p24 capsid immunostaining 72 h later. Importantly, the same compounds found to be effective against the HIV-1 vector were also inhibiting wild-type HIV-1 replication with similar IC_50_ and IC_90_ values (Table 1).

**Table 1:** Antiretroviral activity of isoxazolidine and isoxazole sulfonamides

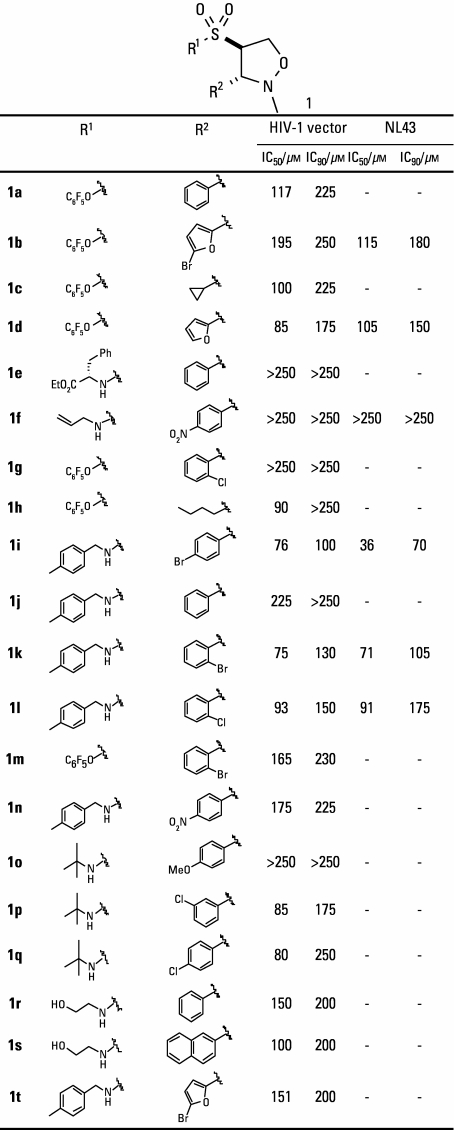

**Figure 1:**
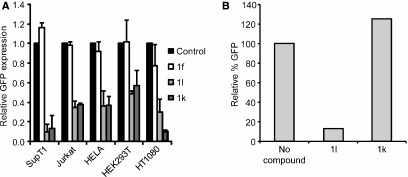
compounds activity in different cell types. (A) The indicated cell types were incubated with the compounds at IC_90_ concentrations for 6 h prior to infection with the HIV-1 vector. Bars show the average percentage of infected cells relative to control (no compound added) ± average deviation. Results were compiled from two independent experiments performed in duplicate. (B) SupT1 cells were incubated with the indicated compounds at IC_90_ concentrations for 6 h prior infection with an MLV vector and analyzed 24 h later by flow cytometry. Similar results were obtained in another independent experiment.

Next, we considered whether the compounds were similarly active in cell types other than CD4+ T cells. To this end, we first tested the compounds on several different cell lines at IC_90_ concentrations, including the epithelial cell line HeLa, the embryonic fibroblastic cell line 293T and the osteosarcoma cell line HT1080. The HIV-1 viral vector was inhibited in all cell types, but the compounds varied in potency in different cell lines (Figure 1A). We also tested compound **1l** on another retrovirus unrelated to HIV, the murine leukemia virus (MLV), and found it to be similarly effective (Figure 1B). The different potency of the compounds in different cell lines and their ability to inhibit replication of two unrelated retroviruses with similar potency hinted at the possibility that the compounds targeted a host factor broadly required for retrovirus replication rather than a viral enzyme.

### Isoxazolidines with antiretroviral activity are not toxic

**Figure 2:**
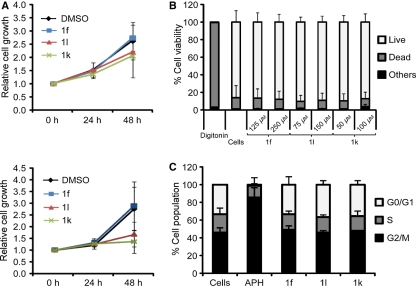
The compounds show no significant cell toxicity at the IC_90_ concentrations. (A) Analysis of cell growth. SupT1 cells were incubated with the compounds at IC_90_ concentrations (*bottom*) and half the IC_90_ concentrations (*top*) and counted every 24 h. (B) Analysis of cell viability. SupT1 cells were incubated with the indicated compounds at IC_90_ concentrations and half the IC_90_ concentrations for 30 h prior to double fluorescent labeling for the detection of live and dead cells by flow cytometry (for details refer to Materials and Methods). *Digitonin,* cells treated with digitonin as a positive control for dead cells. *Cells,* samples incubated with DMSO only. (C) Cell cycle profiling. HeLa cells were incubated with the indicated compounds at IC_90_ concentrations for 30 h prior to staining with PI and flow cytometry. *Cells*, samples incubated with DMSO only. *APH*, cells incubated with aphidicolin. Values were compiled from two independent experiments performed in triplicates.

It was important to assess whether the compounds are toxic to exclude a non-specific antiretroviral effect. Hence, we studied the effect of the compounds on (i) cell growth, (ii) cell viability and (iii) cell cycle progression. To study cell growth, SupT1 cells were incubated with the compounds at IC_50_ and IC_90_ concentrations over a period of 48 h, and the number of viable cells was counted every 24 h. Figure 2A shows that **1l** and **1k** inhibited cell growth at IC_90_ concentrations after 48 h, but at 24 h cell growth was maintained. To examine cell viability, SupT1 cells were exposed to the compounds at IC_90_ and half of the IC_90_ concentrations for 24 h and analyzed by flow cytometry using a double fluorescent assay to label live and dead cells. No loss of cell viability was detected in these conditions (Figure 2B). The same conditions were used to examine cell cycle progression by propidium iodide staining, and aphidicolin, a DNA polymerase inhibitor that arrests cells in the G1/S phase (22), was used as a positive control. No significant changes in the cell cycle profile were detected in cells exposed to the compounds compared to control cells (Figure 2C). Taken together, these results showed that the compounds were not toxic at the IC_90_ concentration for a period of 24 h, and hence their antiretroviral effect was specific. In all further experiments conducted, cells were only exposed to the compounds for 24 h.

### Isoxazolidines inhibit the establishment of an actively transcribing provirus

**Figure 3:**
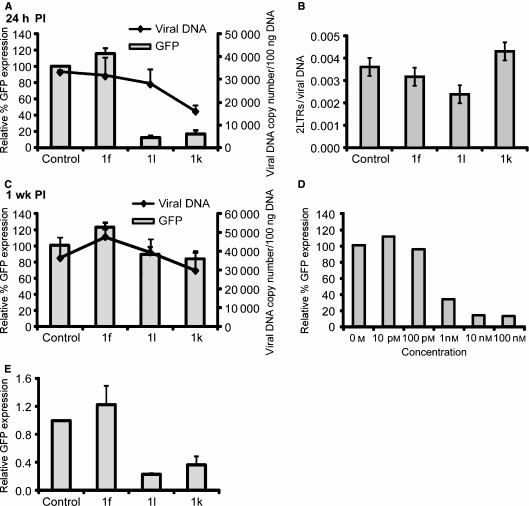
Impact of the compounds on early steps of the HIV-1 infection cycle. (A–C) SupT1 cells were incubated with compounds at IC_90_ concentrations for 6 h prior to infection with an HIV-1 vector, and cells were analyzed 24 h later by flow cytometry and TaqMan qPCR. (A) Quantification of infected (GFP+) cells and viral DNA copies synthesized 24 h post infection. (B) Determination by Taqman qPCR of the ratio of 2LTRs circular DNA to linear viral DNA 24 h post infection. (C) Quantification of infected (GFP+) cells and copies of viral DNA present in cells 1 week post infection. (D) Quantification of infected (GFP+) cells 1 week post-infection; cells were exposed to raltegravir at the indicated concentration for 24h. (E) Viral gene expression is defective in the presence of the compounds. Human 293T cells were incubated with the compounds for 6 h prior to transfection with the HIV-1 plasmid pLAI, which transcribes GFP from the wild-type LTR promoter. GFP expression was detected 24 h post transfection by flow cytometry. Values are expressed relative to control. *Control*, cells incubated with DMSO only. Bars represent the average values ± SE from two independent experiments performed in duplicate.

We investigated the step(s) of the HIV-1 life that were impaired by the small compounds. The HIV-1 life cycle can be simplified into discrete steps such as entry into cells, reverse transcription, nuclear transport, integration into host chromosomes, transcription and exit/budding (23). Exit and budding are generally considered ‘late steps’. Experiments shown in Table 1 already indicated that the compounds impaired some early step in virus replication, because they were equally effective against the HIV-1 vector in single-cycle assays (the vector is unable to exit or bud) and wild-type HIV-1 in replication assays. Furthermore, results shown in Table 1 and Figure 1 also indicated that virus entry into cells was not the main step blocked, because infection was similarly impaired in cells infected with a pan-tropic VSV-G-pseudotyped vector and a wild-type NL4.3 HIV-1, which uses CD4 and CXCR4 as receptor and co-receptor, respectively (24). To examine reverse transcription, cells were infected in the presence of the compounds at IC_90_ concentrations and analyzed by flow cytometry and Taqman qPCR to measure the amount of reverse transcribed viral DNA. Synthesis of viral DNA was not significantly impaired in the presence of **1l** and was reduced by about twofold in the presence of **1k**, which did not account for the much greater inhibition of infection as detected by flow cytometry for GFP expression (Figure 3A). This result confirmed that entry and reverse transcription were not the main steps blocked by the compounds. Furthermore, no reduction in 2LTR circular viral DNA (a hallmark of nuclear entry (25)) relative to total viral DNA was observed by qPCR 24 h post infection with **1f** and **1k,** and a modest reduction was detected with **1l**, suggesting that nuclear transport of the viral complex was not significantly impaired (Figure 3B). To measure HIV-1 integration into host chromosomes, cells infected in the presence of the compounds were serially passaged for 1 week. In these conditions, un-integrated DNA is gradually lost by dilution and degradation (25,26). In parallel experiments, infection was carried out in the presence of Raltegravir, a potent and specific integrase inhibitor (27), which was washed out after 24 h like the other compounds (Figure 3D). No significant reduction of GFP and viral DNA was detected 1 week post infection in cells treated with compounds **1l** and **1k**, as opposed to cells treated with raltegravir in which GFP was reduced in a dose-dependent way (Figure 3). These results indicated that the compounds did not inhibit integration in a detectable way.

**Figure 4:**
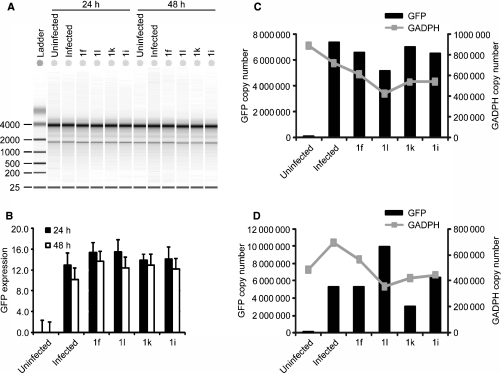
The compounds do not inhibit steady-state levels of transcription. SupT1 cells were infected with HIV-1 LAI Δ env (expressing GFP) pseudotyped with VSV-G, and compounds were added at IC_90_ concentrations 72 h post infection, once steady-state levels of transcription had been reached. Aliquots of cells were analyzed 24 and 48 h after addition of the compounds by flow cytometry (to detect GFP+ infected cells) and RT-qPCR. *Infected*, infected cells incubated with DMSO only. (A) Gel electrophoresis of RNA extracted from cells. RNA concentration in each lane is approximately 100 ng/μL with a minimum RNA integrity of 85%. (B) Quantification of GFP-expressing cells. Bars represent the average percentage of GFP+ cells ± SE after 24 and 48 h of incubation with the compounds. Results were compiled from two independent experiments performed in duplicate. (C) GFP and GADPH mRNA copy number measured by RT-qPCR 24 h after the addition of the compounds. Samples incubated without RT gave undetectable signal. (D) Same as C but analysis was performed at 48 h after the addition of compounds. Results are representative of two independent experiments.

Next, we examined if the compounds impaired viral gene expression. Transfection of cells with a plasmid driving transcription of a marker gene from the HIV-1 LTR is a standard method to examine viral gene expression (28); hence, we transfected cells with pLAI Δ env (a near full-length HIV-1 plasmid with a deletion in env and encoding GFP) (29) in the presence of the compounds and measured gene expression 24 h later by flow cytometry. Remarkably, the compounds inhibited viral gene expression to a level that essentially matched the block to infection (Figure 3E), indicating that this step was severely impaired. To follow up on this observation, cells chronically infected with HIV-1 LAI Δ env were incubated with the compounds for 24 and 48 h, total RNA extracted and viral mRNA quantified by Taqman RT-PCR. An aliquot of the cells was also examined by flow cytometry to detect gene expression at the protein level. Surprisingly, viral mRNA and protein levels were not reduced significantly by the compounds at either time-point (Figure 4). The compounds did not have a significant effect on transcription of a housekeeping gene such as GADPH (Figure 4C and D); hence, they did not broadly repress RNA polymerase II-dependent transcription. These results indicated that the compounds block the establishment of an actively transcribing virus rather than perturbing steady-state transcription, mRNA export or translation of viral proteins.

### Antiretroviral activity depends on specific chemical substitution pattern

**Figure 5:**
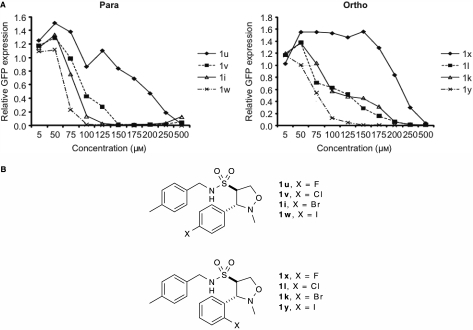
The halogen atom in the aryl group R^2^ determines the antiretroviral potency of the isoxazolidines **1**. (A) SupT1 cells were incubated with varying concentrations of compounds for 6 h prior infection with an HIV-1 vector. The number of GFP+ cells was quantified by flow cytometry 24 h post infection. Values are expressed relative to cells incubated with DMSO only. *Left* shows graph of compounds with the halogen atom in *para* position. *Right* shows graph of compounds with the halogen atom in *ortho* position. Values are representative of two independent experiments. (B) Structures of compounds tested in A.

Based on the data collected from out initial screening, we evaluated a second-generation compound collection based on the most active compounds from our initial screening **1i** and **1k** (Table 1). Whereby, we fixed the C-4 sulfonamide varied the position, *ortho* or *para*, and nature of a halogen substituent, F, Cl, Br or I, in the halobenzene substituent at C-3, R^2^. Interestingly, the size of the halogen atom clearly influenced compounds’ potency (Figure 5). Thus, both iodinated isoxazolidines **1w** and **1y** were the most potent in each regioisomeric series. Furthermore, compounds having the aryl group with a halo-substitution in *para* position, **1u**, **1v**, **1i** and **1w**, were generally more potent than the corresponding *ortho*-substituted isomers (Figure 5).

### Antiretroviral activity of isoxazole sulfonamides

**Figure 6:**
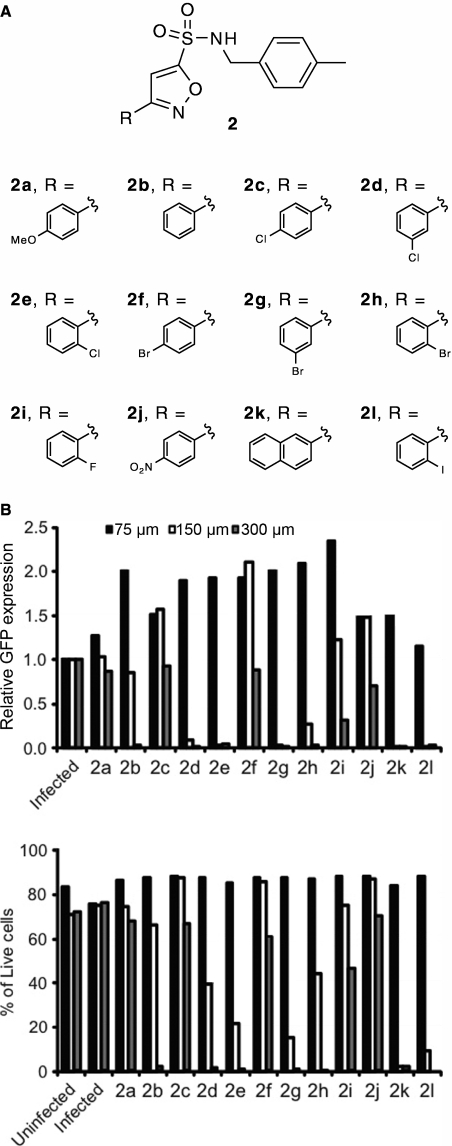
Activity and toxicity of isoxazole sulfonates and sulfonamides. (A) Chemical structures of isoxazole sulfonamides. (BC) SupT1 cells were incubated with three concentrations of compounds for 6 h prior infection with an HIV-1 vector at an MOI of 0.05. The number of GFP+ cells and live cells was quantified by flow cytometry and is shown relative to control. *Un-infected*, cells incubated with DMSO only; *infected*, cells infected in the absence of compound. (B) Proportion of GFP+ cells relative to control (*top panel*) and percentage of live cells (*bottom panel*) for isoxazole sulfonamides **2**.

We then evaluated a related class of isoxazole sulfonamides (series **2**). Based on the results obtained for the isoxazolidines **1,** our library focused on benzyl sulfonamides and isoxazoles bearing halogenated aromatics in the C-3 position. Although some members of this class displayed significant antiretroviral activity, e.g. **2d**, **2g** and **2i**, in general as a class isoxazoles **2** showed a greater cell toxicity and lower antiretroviral activity than the isoxazolidines **1** (Figure 6). We cannot currently, based on this limited data set, attribute the increased antiretroviral activity to a particular structural characteristic of isoxazoles **2**.

### Selection of a HIV-1 mutant partially resistant to the compounds

**Figure 7:**
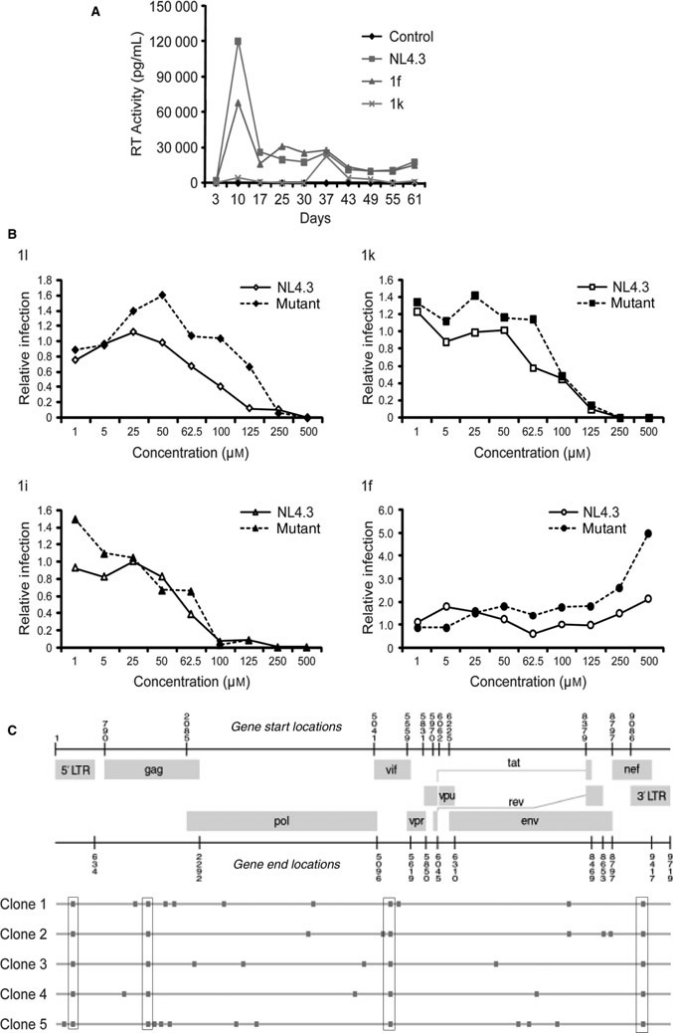
Isolation and characterization of a mutant virus partially resistant to the compounds. (A)C8166 cells were infected with wild-type HIV-1 NL4.3 at an MOI of 0.03 in the presence of an IC_25_ dose of the indicated compounds. RT activity was monitored at regular time intervals to detect any breakthrough virus. *Control*, uninfected cells incubated with DMSO only. *NL4.3,* infected cells incubated with DMSO only. (B) The parental and breakthrough viruses obtained at day 37 were tested in fresh C8166 cells in the presence of different concentrations of the indicated compounds. Cells were analyzed 72 h post infection by flow cytometry. Data are a representative of two independent experiments. (C) Schematic representation of the HIV-1 genome and the position of mutations detected in the partially resistant virus compared to the parental virus. Five clones of the partially resistant virus were sequenced with overlapping primers; *gray dots* indicate the position of point mutations present only in individual clones; *Boxed dots* indicate point mutations found in all five clones.

To better understand the mechanism of action of these compounds, we attempted to select an HIV-1 resistant strain. To this end, wild-type NL4.3 virus was grown in C8166 cells over an extended period of time (61 days) in the presence of compound **1k** at IC_25_ or the inactive compound **1f** at the same concentration. Reverse transcriptase (RT) activity was monitored at regular intervals to detect any virus breakthrough. In a similar assay, viruses resistant to the RT inhibitor nevirapine (30) could be consistently selected (not shown). Virus grown in the presence of DMSO or compound **1f** reached a peak of RT activity after 10 days in culture. A transient spike of RT activity was detected also for the virus grown in the presence of compound **1k** at day 37. Unfortunately, RT activity was gradually lost, although it remained above background for up to 61 days of continuous propagation (Figure 7A). Despite several attempts, a fully resistant virus could not be rescued. The partially resistant virus was retested in independent assays in the presence of increasing concentrations of compounds **1f** (inactive), **1l** (active with an IC_50_ of ∼90 μm), **1k** (active with an IC_50_ of ∼75 μm) and **1i** (active with an IC_50_ of ∼70 μm). Partially resistant and wild-type viruses grew equally well in the presence of compound **1f,** but the partially resistant virus showed a shift in the IC_50_ with compound **1l** (∼90–150 μm) and compound **1k** (∼75–110 μm), but no IC_50_ shift was detected with compound **1i** (Figure 7B). Thus, the selected virus was partially resistant only to the least potent compound but still susceptible to compounds with a larger halogen atom in the aryl group at C-4.

The resistant and the parental viruses were sequenced in parallel, and several mutations were detected. Four mutations were present in all clones analyzed of the partially resistant virus, but not in the parental virus. A search of the Los Alamos HIV-1 repository sequence database confirmed that such mutations were not polymorfic. One mutation was at nt 257 (positions are referred to the HIV-1 NL4.3 reference sequence (19)) in the U3 region of the 5′ LTR, which lies within a DNAse I hypersensitive site upstream of nucleosome nuc-1 (31); a second mutation at nt 1441 caused a neutral change in residue 86 of p24 capsid from valine to methionine; a third mutation at nt 5167 caused a non-neutral change in residue 43 of vif from histidine to tyrosine; and a fourth mutation at nt 9016 caused a non-neutral change in residue 77 of nef from arginine to isoleucine.

## Discussion

We have used novel synthetic methods to obtain sulfonamide derivatives with broad antiretroviral activity. Systematic analysis of the HIV-1 life cycle showed that the small compounds inhibited an early step of the HIV-1 life cycle coincident with the establishment of an actively transcribing virus. It should be noted that the HIV-1 vector transcribes its unspliced mRNA from an internal spleen focus-forming virus (SFFV) promoter, not the wild-type HIV-1 LTRs (16). Because the compounds inhibited equally well the HIV-1 vector and wild-type HIV-1, any inhibition of gene expression mediated by the compounds is bound to be Tat- and Rev-independent. Moreover, the compounds did not broadly inhibit RNA polymerase II transcription, including viral gene transcription, at steady state nor did they suppress protein translation. The simplest explanation for our results is that our compounds interfere with some rearrangements in viral DNA structure that are required for viral transcriptional activation or that they prevent correct assembly of the transcriptional machinery on the viral promoter (31). It is known that the proviral enhancer/promoter within the HIV-1 LTR is organized in precisely positioned nucleosomes and nucleosome-free regions (31). The rapid removal of one nucleosome (nuc-1), positioned at nt 465–610, is a prerequisite for transcriptional induction of the viral promoter (31), suggesting that specific chromatin remodeling may be necessary for provirus activation (32). Therefore, our compounds may target host factors part of the chromatin remodeling machinery that are required for transcriptional activation of several retroviral promoters, and transcriptional activation may be a valid target for antiretroviral drug development (32). Interestingly, a phenotye similar to the one we described here was reported previously and was shown to be caused by repression of the cellular sulfonation pathway (26). In the future, it will be interesting to investigate whether the sulfonamide derivatives described in this study inhibit the sulfonation pathway.

Structure-activity relationship (SAR) studies identified two elements critical for antiretroviral activity in isoxazolidines **1**, an aryl amide at R^1^ and a halogenated benzene at R^2^ (see Table 1). Moreover, the size of the halogen and its position in the C-3 benzene significantly influenced potency. Evaluation of the structurally related isoxazoles **2** revealed compounds with increased cell toxicity and often reduced antiretroviral activity compared to isoxazolidines **1**. By identifying the chemical substituents critical for antiretroviral activity on the one hand and chemical regions and substitutions increasing cell toxicity on the other hand, the SAR results provided a good starting point for further modifications aimed at improving potency and specificity of the compounds.

We attempted to select for an HIV-1 strain resistant to one of the compounds, and we could detect a spike of RT activity after 37 days of continuous propagation in the presence of **1k**. However, the escape virus could not be propagated in the long term, although low-level replication did occur until day 61 as detected by RT enzymatic assay. It is not clear at present why the virus releasing higher RT activity at day 37 could not be further propagated. It could be attributable to some different culture conditions inadvertently introduced or to the fact that the escape virus was less fit in the long term. In any case, a virus was obtained that was partially resistant to the least potent isoxazolidine **1l** but was still fully sensitive to the more potent isoxazolidine **1i**. This suggested that the virus might have evolved a greater affinity for the host factor targeted by the compounds. Sequencing of the partially resistant mutant virus revealed four unique mutations, two of which were non-neutral aminoacid changes, one in vif and one in nef. One mutation was located in the U3 region of the 5′ LTR, which may be interesting because it maps within the DNAse I hypersensitive site upstream of nucleosome nuc-1, which must be removed to allow trascriptional induction (31). It will be interesting in the future to introduce by site-directed mutagenesis such mutations one by one or in combination into a wild-type HIV-1 NL4.3 molecular clone to examine their physiological role. Although longer-term virus passaging is required to further explore this aspect, these results suggest that small compounds targeting host factors may be less vulnerable to the development of resistance.

In conclusion, using novel synthetic approaches, we have generated new sulfonamide derivatives able to block transcriptional activation of HIV-1 and determined the chemical regions and modifications important for their antiretroviral activity. The availability of active and inactive compounds with very similar chemical structures should allow the identification of the target and lead to further improvement of potency and specificity (13). It may also reveal novel druggable host factors important for HIV-1 replication.

## Note

a UNAIDS (2008) Report on the global AIDS epidemic. http://www.unaids.org.
